# Using mortality follow-up of surveys to estimate social inequalities in healthy life years

**DOI:** 10.1186/1478-7954-12-13

**Published:** 2014-05-12

**Authors:** Rana Charafeddine, Nicolas Berger, Stefaan Demarest, Herman Van Oyen

**Affiliations:** 1 Scientific Institute of Public Health, Operational Direction Public Health and Surveillance, 14, Juliette Wytsmanstreet, Brussels 1050, Belgium

**Keywords:** Healthy life years, Socioeconomic status, Surveys, Mortality follow-up, Monitoring

## Abstract

**Background:**

The estimation of healthy life years (HLY) by socio-economic status (SES) requires two types of data: the prevalence of activity limitation by SES generally extracted from surveys and mortality rates by SES generally derived from a linkage between the SES information in population databases (census, register) and mortality records. In some situations, no population-wide databases are available to produce mortality rates by SES, and therefore some alternatives must be explored. This paper assesses the validity of calculating HLY by SES using mortality rates derived from a linkage between surveys and mortality records.

**Methods:**

Two surveys were chosen to explore the validity of the proposed approach: The Belgian Health Interview Survey (HIS) and the Belgian Survey on Income and Living Conditions (SILC). The mortality follow-up of these surveys were used to calculate HLY by educational level at age 25. These HLY were compared with HLY estimates calculated using the mortality follow-up of the 2001 census. The validity of this approach was evaluated against two criteria. First, the HLY calculated using the census and those calculated using the surveys must not be significantly different. Second, survey-based HLY must show significant social inequalities since such inequalities have been consistently reported with census-based HLY.

**Results:**

Both criteria were met. First, for each educational category, no statistically significant difference was found when comparing census-based and survey-based HLY estimates. For instance, men in the lowest educational category have shown a HLY of 34 years according to the HIS, and while this figure was 35.5 years according to the census, this difference was not statistically significant. Second, the survey-based HLY have shown a significant social gradient. For instance, men in the highest educational category are expected to live 9.5 more HLY than their counterparts in the lowest educational category based on the HIS estimates, compared with 7.3 HLY based on the census estimates.

**Conclusions:**

This article suggests that using the mortality follow-up of a nationally representative cross-sectional survey is a valid approach to monitor social inequalities in HLY in the absence of population-wide data.

## Background

Health expectancies (HE) are summary measures of population health that combine length and quality of life into a single measure. They are used for a variety of research and policy purposes such as highlighting health inequalities and evaluating health policies [1]. HE refer to an entire class of indicators expressed in terms of life expectancy in a given state of health. One of the most commonly reported HE is the healthy life years (HLY) indicator that measures the number of remaining years that a person of a certain age is expected to live without activity limitation. The European Union (EU) has selected HLY to be among the core set of the indicators to be monitored annually by Eurostat. The importance of this indicator was recognized in the Lisbon Strategy and in the current EU 2020 strategy for the assessment of social policies in relation to retirement age, health care costs, and long term care for the aging population [2].

Efforts have been dedicated at the European level to ensure comparability of the HLY estimations across EU Member States and to establish a sustainable monitoring system. These efforts are aimed mainly at ensuring the monitoring of HLY at the population level, but currently there are increasing requests to estimate this indicator by population subgroups, notably socio-economic groups [3]. Social inequalities in HE have been widely documented [4-7]. For instance, using Belgian data, Van Oyen and colleagues [7] reported that those with a higher level of education live longer and live fewer years with disability compared with those with a lower educational level. Analyzing the evolution of these inequalities over time, the authors concluded that the educational gradient has increased in the country between 1997 and 2004. This highlights the importance of monitoring these indicators not only at the population level, but also by socio-economic groups.

To compute HLY, the most widely used approach is the Sullivan method [8]. This approach requires two types of data: 1) age- and sex-specific mortality rates taken from a period life table and 2) age- and sex-specific prevalence of activity limitation taken from a cross-sectional survey. To calculate social inequalities in HLY, such data are needed for the different socio-economic categories under study. While data about the prevalence of activity limitation by socio-economic status (SES) can be obtained through health surveys, mortality data by SES are not routinely produced. Generally, mortality data by SES are extracted from databases linking population-wide data (census, registers) with mortality records [9,10]. A nonsystematic review of approaches used to estimate mortality by SES in a number of European countries is found in Additional file 1.

In Belgium, data on activity limitation by SES are available from surveys such as the Health Interview Survey (HIS) or the Statistics on Income and Living Conditions Survey (SILC). However, the availability of mortality rates by SES is more problematic. In the past, such information was produced using the mortality follow-up of the national census. For instance, Van Oyen et al. [7] and Deboosere et al. [10] used the mortality follow-up of the 1991 and 2001 censuses. For the 2011 census there was a decision to replace the census where data are collected via a questionnaire by an administrative census where the data are compiled from existing data sources. It is not clear to what extent this administrative census will be effective for estimating HLY by SES, since it has not yet been released as of February 2014. For instance, it is not certain that information for immigrants and for those who have received their diploma outside Belgium will be accurately included in the database. In this context, an alternative to the census must be explored. Surveys are already used to estimate the activity limitation part of the HLY, therefore a proposal can be made to use the mortality follow-up of the same surveys to estimate the mortality component. Such an approach may have a non-negligible impact on the HLY estimates, as a survey and a census differ in a number of ways. A census collects information about every member of a given population, while a survey collects information about a sample of a given population. A census is compulsory, while people may choose not to participate in a survey. As a result, census data are more accurate and reliable than survey data, since nonparticipation to surveys may introduce a selection bias. A census is, however, very demanding in terms of resources, which led a number of countries to find alternatives to the census data collection effort. In this context, the present article aims to investigate the validity of using the mortality follow-up of surveys as a substitute to the use of the mortality follow-up of the census to estimate and monitor social inequalities in HLY in Belgium.

## Methods

### Data

Two surveys were selected to extract activity limitation data and mortality rates by SES: the HIS of 2001 and the SILC of 2004. More details on these surveys are found in Table 1 and the methodological approaches are described elsewhere [11,12]. A record linkage was undertaken between these surveys and the National Register using a unique identifier present in both data sources. The authorization for such a linkage is regulated by the Privacy Commission. After the approval of the Commission, the following data were obtained: 1) mortality follow-up for the HIS 2001 until 31/12/2010 and 2) mortality follow-up for the SILC 2004 until 31/12/2009. The household response rate is 61.3% for the HIS 2001 and 48.6%^1^ for the SILC 2004. The linkage was successful for 97% of the HIS records and for 96% of the SILC records. The final HIS sample included 8,510 individuals aged 15 years and older and 1,034 deaths, and the SILC included 9,312 individuals aged 16 years and older and 435 deaths.

**Table 1:** General description of the Health Interview Survey (HIS) and the Survey on Income and Living Conditions (SILC), Belgium

**Criteria**	**HIS**	**SILC**
Aim of the survey	To describe the health status of the population and monitor health trends in Belgium and its three regions.	To provide a complete set of indicators on poverty, social exclusion, pensions, and material deprivation.
Health indicators	A wide series of health indicators, including the Global Activity Limitation Indicator (GALI) used to estimate HLY.	Three generic health indicators including the Global Activity Limitation Indicator (GALI).
Socio-economic status indictors	Education, income, employment.	Education, income, employment.
Mortality follow-up	Linkage with mortality data of the National Register using a unique identifier is possible after privacy commission approval.	Linkage with mortality data of the National Register using a unique identifier is possible after privacy commission approval.
Survey design	Stratified multistage clustered scheme.	Stratified two-stage sampling scheme in 2004, followed by rotation since 2005. Rotation allows to replace roughly one-fourth of the sample each year. The rotation pattern is such that the overlap between samples in any two successive years is roughly 75%, and that the sample is completely renewed after four years.
	The sample is representative at the national and regional levels.	The sample is representative at the national level.
Sample size	Approximately 10,000 individuals, although in some years the sample is bigger due to an oversampling of some age groups or municipalities.	Approximately 9,000 individuals .
Target population	All individuals residing in Belgium at the time of data collection. The GALI used to estimate HLY is included in the self-completed questionnaire that is given only to those aged 15 years and older.	All individuals residing in Belgium at the time of data collection aged 16 years and older.
Response rate	For 2008, the household response rate was about 55%.	For 2010, the household response rate was 43.3% for new households (households drawn for the first time in 2010) and 63.5% when accounting for the new and old households (households that contain at least one person who took part in 2009 and had to be surveyed again in 2010 according to the rotation design).
Periodicity	Every three to five years.	Annual.

The HLY calculated using these surveys will be compared with the HLY calculated using the 2001 census. The latter were estimated using the mortality by SES information based on the linkage between the 2001 census and the mortality records for the period 2001–2004, which were derived from the work of Deboosere et al. [10]. The record linkage was undertaken using a unique identifier present in both the census and the National Register.

### Measures

#### Socioeconomic status

Educational level was selected as an indicator of SES and reflects the highest educational level achieved by the individual. In both surveys, the indicator was recoded as primary educational level, lower secondary, higher secondary, and higher education. Individuals categorized as having no diploma have been dropped from the analysis to allow comparison with the census-based calculations of Deboosere et al. [10], which treated separately those with no diploma and those with a primary educational level. The number of people with no diploma is, however, too low in the SILC and in the HIS to allow a separate analysis.

#### Activity limitation

The HIS and the SILC include similar generic information on activity limitation based on the Global Activity Limitation Indicator (GALI). For the HIS, the formulation is as follows: “For the past 6 months or more have you been limited in activities people usually do because of health problem? Yes strongly limited, Yes limited, No, not limited.” For the SILC, it is: “For at least the past 6 months, to what extent have you been limited because of a health problem in activities people usually do? Would you say you have been? Severely limited, Limited but not severely, Not limited at all.”

#### Healthy life years

HLY at age 25 were calculated using the Sullivan method [13,14]. This method is based on the life table, which describes the survival experience of a real or hypothetical group of people followed from birth or from other ages in their lifetime. The life table is then combined with the age-specific prevalence of activity limitation to estimate HLY [13,14]. A brief description of the calculation process is as follows:

First, the mortality follow-up of the HIS and the SILC were used to derive the age-specific person-years. This was done while accounting for the age change during the follow-up period with a procedure called Lexis expansion where the observed individual follow-up times were split into periods that correspond to different current-age (or attained-age) groups [15]. Therefore, each subject’s person-years of observation were split into several observations by expanding data by one-year age bands. The age-specific person years and the number of deaths by age group were then used to calculate total life expectancy (LE).

Second, the age specific prevalence rates of activity limitation based on the HIS and the SILC were integrated in the life tables to calculate the total number of years lived with activity limitation and the total number of years lived without activity limitation (HLY). The activity limitation prevalence rates were weighted to account for the study design of the specific survey.

Third, the standard error of the HLY was approximated. The total standard error is composed of a part due to the prevalence rates and a part due to the mortality rates. The part of the variance due to the mortality rates can be ignored when using the mortality follow-up of census data, but it has to be accounted for when using the mortality follow-up of surveys.

### Analytic approach

Two criteria were used to assess the validity of using the mortality follow-up of a survey to estimate social inequalities in HLY.

1. The difference between the HLY calculated using the surveys and the HLY calculated using the census must not be statistically significant (α = 0.05). We used a z-test to assess the difference between two comparable HLY estimates calculated based on mortality rates from two different sources: one based on mortality rates generated using a three-year follow-up of the 2001 census(census-based HLY), and the other based on the mortality follow-up of the surveys (survey-based HLY, HIS-based HLY, SILC-based HLY). The calculation of the census-based HLY differs from the survey-based HLY in two ways: 1) The life table was based on the mortality follow-up of the census and not on the surveys. 2) The standard error approximation took into account only the variance due to the prevalence rates, as there is no uncertainty concerning the mortality rates derived from the census. For both HLY estimates, the activity limitation portion used was similar.

2. Social inequalities in survey-based HLY must be statistically significant (α = 0.05), since such inequalities have been consistently reported in the literature with health expectancies calculated using population-based datasets [4,7]. Significant social inequalities in HLY were determined using a z-test that assesses the difference in HLY between the lowest and the highest educational categories [14].

## Results

Table 2 displays the prevalence of activity limitation by sex and educational level using the HIS and the SILC. Both surveys reveal substantial educational inequalities, where those with a higher educational level have lower prevalence of activity limitation.

**Table 2:** Prevalence of activity limitation by sex and educational level for the HIS 2001 and SILC 2004, Belgium

**Survey**	**Males**			**95%**	**Females**			**95%**
	**Education**	**N**	**Weighted %**	**Confidence Interval**	**Education**	**N**	**Weighted %**	**Confidence Interval**
HIS	Primary education	601	36.2	31.2-41.2	Primary education	800	41.4	36.9-45.8
	Lower secondary	902	24.2	20.6-27.8	Lower secondary	954	25.0	21.2-28.7
	Higher secondary	1385	14.1	11.7-16.6	Higher secondary	1288	19.1	16.1-22.0
	Higher education	1250	13.2	10.7-15.8	Higher education	1330	10.3	8.2-12.3
	Total	4138	19.0	17.5-20.6	Total	4372	21.8	20.1-23.4
SILC	Primary education	615	39.2	35.1-43.4	Primary education	805	50.9	47.2-54.5
	Lower secondary	841	27.2	24.1-30.4	Lower secondary	849	35.3	31.9-38.7
	Higher secondary	1837	22.3	20.3-24.3	Higher secondary	1656	25.1	22.9-27.4
	Higher education	1296	18.5	16.0-20.9	Higher education	1413	19.4	17.2-21.6
	Total	4589	24.4	23.1-25.7	Total	4723	29.9	28.5-31.3

Table 3 presents the age-adjusted mortality rates by sex and educational level for both surveys. The figures show that the higher the level of education, the lower the mortality rate. In the HIS, for instance, the mortality rate among men with a primary educational level is 1056.4 per 100,000 person years (PY), while this figure is 495.1 per 100,000 PY for men with a higher education. In the SILC, these figures are 1123.67 per 100,000 compared with 452.43 per 100,000.

**Table 3:** **Age-adjusted mortality rate**^1 ^**per 100,000 person-years by sex and educational level for the HIS 2001-2010 and SILC 2004-2009, Belgium**

**HIS**	**Person-years**	**Mortality rates**	**Lower 95% CI**	**Upper 95% CI**
**Education**				
**Males**				
Primary education	9253	1056.4	783.4	1329.3
Lower secondary	11058	760.7	587.8	933.7
Higher secondary	14887	689.9	526.5	853.3
Higher education	12825	495.1	342.9	647.3
**Total**	48023	715.7	633.9	797.5
**Females**				
Primary education	11340	565.0	421.5	708.5
Lower secondary	11541	430.9	307.6	554.1
Higher secondary	14023	372.8	248.8	496.9
Higher education	13867	296.9	112.9	480.9
**Total**	50771	437.4	365.2	509.6
**SILC**	**Person-years**	**Mortality rates**	**Lower 95% CI**	**Upper 95% CI**
**Women**				
**Males**				
Primary education	3412	1123.7	811.2	1436.2
Lower secondary	4803	942.0	649.7	1234.4
Higher secondary	10731	792.6	595.2	990.1
Higher education	7570	452.4	293.7	611.1
**Total**	26516	796.1	688.0	904.1
**Females**				
Primary education	4353	502.8	328.8	676.8
Lower secondary	4969	383.0	223.0	542.9
Higher secondary	9790	432.6	279.7	585.6
Higher education	8279	287.4	149.2	425.5
**Total**	27391	423.6	346.9	500.3

^1^The 1976 European Standard Population is used as a standard population [16].

Table 4 and 5 show survey-based and census-based life expectancy (LE) and HLY by educational level among males (Table 4) and females (Table 5) aged 25 years. For instance, based on the HIS estimations, 25-year-old males with the lowest educational achievement have a LE of 46.7 years, and out of these years, 34 are expected to be without activity limitation. For the same group, the census-based estimations show a LE of 49.5 years and a HLY of 35.5 years. For all educational categories, no significant differences are detected between census-based and HIS-based estimations. For instance, the difference in LE between these two data sources for 25-year-old males with the lowest educational achievement is 2.8 years. For HLY, this difference amounts to 1.6 years and is not statistically significant (p = 0.72). Similar trends are detected among females and when comparing census and SILC-based estimates.

**Table 4:** Comparison of the life expectancy (LE) and healthy life years (HLY) calculated using mortality rates based on the Census 2001-2004, the HIS 2001-2010, and the SILC 2004-2009, males, aged 25 years, Belgium

**Males**	**Mortality HIS/Morbidity HIS**			**Mortality Census/Morbidity HIS**			**Diff HIS-Census**	
**Education**	**LE**	**HLY**	**HLY 95%**	**LE**	**HLY**	**HLY 95%**		
			**Confidence interval**			**Confidence interval**	**LE**	**HLY (p-value)**
Primary education	46.7	34.0	30.4-37.5	49.5	35.5	33.5-37.6	-2.8	-1.6 (0.72)
Lower secondary	51.7	36.6	34.6-38.6	51.3	36.5	35.0-38.0	0.4	0.1 (0.96)
Higher secondary	54.3	43.1	41.1-45.1	52.5	41.8	40.4-43.2	1.8	1.3 (0.40)
Higher education	56.3	43.5	41.4-45.6	55.1	42.8	41.2-44.5	1.2	0.7 (0.72)
**Difference highest-lowest (p-value)**	9.6	9.5 (p < 0.05)		5.6	7.3 (p < 0.01)			
Source of data: mortality follow-up of the HIS 2001-2010 and mortality follow-up of the Census 2001-2004								
**Males**	**Mortality SILC/Morbidity SILC**			**Mortality Census/Morbidity SILC**			**Diff SILC-Census**	
**Education**	**LE**	**HLY**	**HLY 95%**	**LE**	**HLY**	**HLY 95%**		
			**Confidence interval**			**Confidence interval**	**LE**	**HLY (p-value)**
Primary education	49.9	31.7	28.6-34.8	49.5	31.3	28.7-34.0	0.4	0.4 (0.94)
Lower secondary	50.7	34.2	31.2-37.2	51.3	34.7	32.8-36.6	-0.6	-0.5 (0.90)
Higher secondary	53.0	38.0	36.0-39.9	52.5	37.6	36.4-38.9	0.5	0.4 (0.82)
Higher education	58.1	44.7	42.4-47.1	55.1	42.6	41.2-44.1	3.0	2.1 (0.30)
**Difference highest-lowest (p-value)**	8.2	13.0 (p < 0.01)		5.6	11.3 (p < 0.01)			
Source of data: mortality follow-up of the SILC 2004-2009 and mortality follow-up of the Census 2001-2004								

**Table 5:** Comparison of the life expectancy (LE) and healthy life years (HLY) calculated using mortality rates based on the Census 2001-2004, the HIS 2001-2010, and the SILC 2004-2009, females, aged 25 years, Belgium

**Females**	**Mortality HIS/Morbidity HIS**			**Mortality Census/Morbidity HIS**			**Diff HIS-Census**	
**Education**	**LE**	**HLY**	**HLY 95%**	**LE**	**HLY**	**HLY 95%**		
			**Confidence interval**			**Confidence interval**	**LE**	**HLY (p-value)**
Primary education	56.2	33.5	30.8-36.2	56.2	33.7	31.3-36.0	0.0	-0.2 (0.96)
Lower secondary	57.3	40.9	39.0-42.9	58.0	41.3	39.6-43.0	-0.7	-0.4 (0.82)
Higher secondary	58.6	42.1	39.7-44.4	58.5	42.0	39.9-44.0	0.1	0.1 (0.97)
Higher education	61.6	49.8	46.9-52.6	59.9	48.6	46.3-50.8	1.7	1.2 (0.73)
**Difference highest-lowest (p-value)**	5.4	16.3(p < 0.01)		3.7	14.9 (p < 0.01)			
Source of data: mortality follow-up of the HIS 2001-2010 and mortality follow-up of the Census 2001-2004								
**Females**	**Mortality SILC/Morbidity SILC**			**Mortality Census/Morbidity SILC**			**Diff SILC-Census**	
**Education**	**LE**	**HLY**	**HLY 95%**	**LE**	**HLY**	**HLY 95%**		
			**Confidence interval**			**Confidence interval**	**LE**	**HLY (p-value)**
Primary education	58.1	30.8	28.0-33.6	56.2	30.0	27.4-32.6	1.9	0.8 (0.83)
Lower secondary	61.5	37.8	35.2-40.4	58.0	35.7	33.6-37.7	3.5	2.1 (0.47)
Higher secondary	59.1	40.2	37.9-42.5	58.5	39.6	38.0-41.3	0.6	0.6 (0.79)
Higher education	61.9	45.4	42.5-48.3	59.9	44.0	42.0-46.1	2.0	1.4 (0.68)
**Difference highest-lowest (p-value)**	3.8	14.6 (p < 0.01)		3.7	14.0 (p < 0.01)			
Source of data: mortality follow-up of the SILC 2004-2009 and mortality follow-up of the Census 2001-2004								

LE and HLY show substantial inequalities by educational level regardless of the data source used to estimate mortality rates. For instance, HIS-based estimations show that at 25 years of age, men in the highest educational category are expected to live 9.6 more years and 9.5 more HLY (p < 0.05) than their counterparts in the lowest educational category. According to the census-based estimations, there are 5.6 years of difference in LE and 7.3 years of difference in HLY (p < 0.05). Similar trends are detected among females and when comparing census and SILC-based estimates.

## Discussion

Two criteria were proposed to assess the validity of using the mortality follow-up of surveys to monitor social inequalities in HLY in Belgium. The first criterion assesses the difference between census- and survey-based HLY estimates for each educational category. Comparing these estimates, our findings have shown no statistically significant difference. The second criterion relates to the detection of statistically significant social inequalities in the survey-based HLY estimates as the literature have consistently shown such inequalities with census-based estimations. Our findings detected differences in HLY between the highest and the lowest educational levels in a comparable, yet larger, scale to the census-based estimates.

Although no statistically significant difference was detected between survey- and census- based HLY, the differences in the calculated LE and HLY are not negligible. This can be due to a potential selection bias in population surveys. Numerous studies have examined the selection bias in surveys and have shown that survey participation is associated with a number of variables including gender, SES, and health status. Survey participants tend to be female, have higher SES, and be healthier compared with their nonparticipating counterparts [17,18]. If this was the case in our study, we would have minimal differences between census-based estimates and survey-based estimates for females and for those with the highest educational level. However, no such pattern emerges in these estimates. On the contrary, those with the highest educational category have consistently quite different estimates when using surveys compared with the census. Similarly, differences between the census- and the survey-based estimates are higher among females in the SILC compared with males. Therefore, although it is acknowledged that there is a potential selection bias in the HIS and the SILC, the extent and direction of this bias is not clear.

Based on the above, this study suggests that using the mortality follow-up of repeated cross-sectional surveys is a feasible alternative to monitor social inequalities in HLY in Belgium when no census- or register-based data are available. It should be highlighted that the HLY estimates are not interchangeable between surveys. In other words, it is possible to compare HLY within surveys but not between surveys. The choice of the survey depends on the purpose of the monitoring. For instance, in Belgium the HIS can be used if the objective is to monitor regional differences in social inequalities, as the HIS is representative at the national and regional levels. When the monitoring system requires annual estimates, then the SILC must be chosen as the SILC data are collected annually, while the HIS data are collected every three to five years.

Another approach to estimating social inequalities in mortality in the absence of a mortality follow-up of the census would be to rely on cross-sectional unlinked data. This entails that mortality data, including the SES of the deceased, are extracted from death certificates while population data, including their SES, are extracted from the census or census based population estimates. Using such data can produce potentially biased estimations due to the lack of comparability of the SES information of the death certificate (reported by a proxy informant, usually a relative of the deceased) with that of the census (self-reported by the person); this is the so-called numerator-denominator bias. Studies examining the magnitude of the numerator-denominator bias have often provided contradictory results [19]. While some studies found substantial differences between the SES data self-reported in the census and data reported by a proxy in the death registry, others found no significant differences between these two data sources. In Belgium, a study was conducted to assess the numerator-denominator bias through the comparison of educational inequalities in mortality rates calculated using the linked (mortality follow-up of the last available census (2001)) and the unlinked approaches for the Brussels-Capital Region [20]. The findings showed that the use of unlinked data results in a considerable bias in the Belgian setting.

The EU published a methodology note that explored the development of a harmonized EU system of data collection and compilation on mortality and SES to enable the publication of comparable statistics on social inequalities in mortality [21]. The authors recommend following a prospective approach linking census data with mortality register data using a single and unique person identity number. To date, this is feasible only in certain EU countries. When no unique identifier is available, the authors recommend the use of deterministic or probabilistic linkage approaches^2^. Two other options were also mentioned: the unlinked design and the design focusing on small areas within a country as the unit of data collection. These two options were described as yielding to potentially biased estimates. The approach explored in this research is intermediate. It uses cross-sectional survey data linked to mortality register data via a single and unique personal identifier. This approach may be promising to produce comparable statistics at the European level if the surveys used are harmonized across countries [22]. The EU-SILC is billed as a comparative cross-national dataset. However, this survey exhibits large disparities in survey methods between countries, which could result in considerable risks on comparability. For instance, the choice of the interview procedure differs across countries, as participating countries are allowed to gather the information through interview surveys (face-to-face or telephone) of a population sample or by using national statistical sources that meet Eurostat data-quality criteria. Therefore, further efforts are needed to explore the validity of using the EU-SILC across countries to produce comparable HLY estimates.

One limitation of this study is related to the different periods covered by the surveys under analysis, which could jeopardize the comparison. The mortality follow-up of the HIS 2001-2010 and SILC 2004-2009 were compared with the mortality follow-up of the national census 2001-2004. These survey years were chosen as the SILC 2004 was the first SILC survey in Belgium, while no mortality follow-up of the HIS 2004 was available.

## Conclusion

Individual linkage between population data (i.e., census or nationwide SES registers) and mortality records is the optimal approach to estimate social inequalities in mortality and in HLY. In the absence of such population datasets, other approaches need to be identified due to the importance of this indicator in the European policy context. This article suggests that using the mortality follow-up of a nationally representative cross-sectional survey is a sound alternative. Future research are needed to examine the impact of the selection bias on the survey-based estimates.

## Endnotes

^1^The response rate for subsequent years is higher (about 60%) –see Table 1 for more details.

^2^The deterministic linkage is applied when records in two databases are linked based on the exact correspondence of values in a set of variables present in both databases. The probabilistic linkage approach creates links between two databases based on a calculated statistical probability of a set of common variables. The probability is used to determine whether a pair of records approximately refers to the same individual.

## Competing interests

The authors declare that they have no competing interests.

## Authors’ contributions

RC drafted the manuscript. HVO developed the study concept. RC and NB performed data analysis. SD supervised the data linkage. HVO, NB, and SD critically reviewed the manuscript. All authors read and approved the final manuscript.

## Supplementary Material

Additional file 1 Approaches used to estimate mortality by SES in a number of European countries. Click here for file
