# Supplementary material for: Using mortality follow-up of surveys to estimate social inequalities in healthy life years
Source: Popul Health Metr. 2014 May 12;12:13. doi: 10.1186/1478-7954-12-13 (PMC4030465; doi:10.1186/1478-7954-12-13)
Supplement: Additional file 1 — Approaches used to estimate mortality by SES in a number of European countries. [file 1478-7954-12-13-S1.doc]

**Additional file 1**

Approaches used to estimate mortality by SES in a number of European countries

| **Country** | **SES indicator** | **Period** | **Source of data** | **Linkage approach** |
| --- | --- | --- | --- | --- |
| **Austria**  Klotz, J., & Doblhammer, G. (2008). Trends in educational mortality differentials in Austria between 1981/82 and 2001/2002: A study based on a linkage of census data and death certificates. *Demographic Research*, *19* 1759-1780. | Education | 1981-1982  1991-1992  2001-2002 | Mortality follow-up of the national census | Individual linkage , no unique personal identifier  Mortality rates by SES were generated through the linkage of individual records of the censuses 1981, 1991, and 2001 with death certificates in a 12-month follow-up period. No ‘direct’ matching through the national register number could be used. Instead, data were matched using primary matching variables: sex, date of birth, and last residential address of the deceased. The overall merging rate was about 90% for the 1981 and the 1991/92 death records, compared with almost 94% for the 2001/2002 death records. |
| **Bulgaria**  Kohler, I. V., Martikainen, P., Smith, K. P., & Elo, I. T. (2008). Educational differences in all-cause mortality by marital status - Evidence from Bulgaria, Finland and the United States. Demographic Research, 19 2011-2042. | Education | 1992-1998 | Mortality follow-up of national census | Individual linkage, unique identifier  In Bulgaria, data for estimating mortality by SES come from a linkage between the 1992 population census and death certificates. The follow-up period stopped in 1998.The link between census and mortality data was performed on the basis of a personal identification number that is uniquely assigned to each Bulgarian citizen and is included in death and census records. Approximately 93 % of all death certificates for the study period were linked to the 1992 census records. |
| **Denmark**  Bronnum-Hansen, H., & Baadsgaard, M. (2012). Widening social inequality in life expectancy in Denmark. A register-based study on social composition and mortality trends for the Danish population. [BMC Public Health](http://www.scopus.com.vdicp.health.fgov.be:8080/source/sourceInfo.url?sourceId=19621&origin=recordpage), Volume 12:994, Issue 1. | Education  Income | 1987-2011 | Linkage of different registers | Individual linkage, unique identifier  In Denmark, a unique personal identification number assigned to  all Danish citizens make it possible to link various register data at the individual level such as successive information on education, income and vital status.To calculate mortality rate by social status, SES data from the registers are linked to mortality records for all inhabitants. |
| **Finland**  Kohler, I. V., Martikainen, P., Smith, K. P., & Elo, I. T. (2008). Educational differences in all-cause mortality by marital status - Evidence from Bulgaria, Finland and the United States. Demographic Research, 19 2011-2042. | Education | 1994-1998 | Mortality follow-up mortality of a sample of the national census | Individual linkage, unique identifier  Census and mortality data were linked using a unique personal identification code. Non-linkage of death records to census records is less than 0.5%. The difference with Bulgarian method is that an 11% random sample of longitudinal census data file was studied in the case of Finland. |
| **France**  Saurel-Cubizolles, M. J., Chastang, J. F., Menvielle, G., Leclerc, A., & Luce, D. (2009). Social inequalities in mortality by cause among men and women in France. Journal of Epidemiology and Community Health, 63(3), 197-202. | Education  Occupation | 1990-1999 | Mortality follow-up of the permanent demographic sample | Individual linkage, unique identifier  The data used in France come from a permanent demographic sample currently including about one million people (approximately 1% of the population), randomly selected. Socio-demographic data are updated at each census and information regarding mortality is permanently updated by the means of vital status forms. In other words, a follow-up of the representative sample is constantly ensured. |
| **Lithuania**  Shkolnikov, V. M., Jasilionis, D., Andreev, E. M., Jdanov, D. A., Stankuniene, V., & Ambrozaltiene, D. (2007). Linked versus unlinked estimates of mortality and length of life by education and marital status: Evidence from the first record linkage study in Lithuania. Social Science & Medicine, 64(7), 1392-1406 | Education | 2001-2004 | Mortality follow-up of the national census | Individual linkage, unique identifier  Linkage between the 2001 census, death and migration records was accomplished using personal identification numbers as unique identifiers for the same individuals. The follow-up ended in 2004. The method allowed matching about 95% of the death records to the corresponding census records. A special redistribution procedure was applied to the remaining census-unlinked deaths. |
| **Netherlands (Eindhoven)**  van Oort, F. V. A., van Lenthe, F. J., & Mackenbach, J. P. (2005). Material, psychosocial, and behavioural factors in the explanation of educational inequalities in mortality in the Netherlands. Journal of Epidemiology and Community Health, 59(3), 214-220. | Education | 1991-1998 | Mortality follow-up of the GLOBE longitudinal survey | Individual linkage, unique identifier  Socio-demographic data were collected within the framework of the prospective GLOBE study (Gezondheid en LevensOmstandigheden Bevolking Eindhoven en omstreken). In 1991 a random sample of 27 070 non-institutionalised Dutch persons (aged 15-74 years) drawn from municipal population registers from Eindhoven and its surrounding was sent a postal questionnaire. In the study a total 4087 respondents were eligible for the analyses. The follow-up of all the subjects started in 1991 and ended in 1998. Information about mortality (and changes of address) was collected annually via municipal population registers and could therefore be easily linked to socio-demographic data. |
| **Spain (Madrid, Barcelona)**  Borrell, C., Regidor, E., Arias, L. C., Navarro, P., Puigpinos, R., Dominguez, V., & Plasencia, A. (1999). Inequalities in mortality according to educational level in two large Southern European cities. International Journal of Epidemiology, 28(1), 58-63. | Education | 1993-1994 | Mortality follow-up of the municipal census | Individual linkage, unique identifier  Mortality rates by SES were estimated in the two major cities of the country: Madrid and Barcelona. The population at risk comes from the 1991 Municipal Censuses. The censuses information is revised every 5 years through the active collection of data by the statistical office of each municipality. Between these major revisions, the information is continually updated to incorporate data on births and deaths. Death register and municipal censuses are thus linked. Using 1993 and 1994 mortality data, linkage problems (due to problems of record linkage or missing data) appeared in 1.5% of deaths in Madrid and 9% in Barcelona. |
| **Switzerland**  Faeh, D., & Bopp, M. (2010). Educational inequalities in mortality and associated risk factors: German- versus French-speaking Switzerland. BMC Public Health, 10. | Education | 1990-2000 | Mortality follow-up of the Swiss National Cohort longitudinal study | Individual linkage , no unique personal identifier  In Switzerland, recent mortality studies by SES are based on the Swiss National Cohort, a national longitudinal research platform. Initial data comes from the 1990 census. Although no unique personal identifier is available in Switzerland, the exact data of birth was included in that census for the first time, making it possible to link census data and mortality data from that period of time onwards. Accordingly, the 1990 census was linked with the 2000 census, mortality and emigration records using both deterministic and probabilistic methods of matching. In the first step, pairs of records that were matched on sex, date of birth, marital status, nationality, religion and place of residence were identified. In subsequent steps, probabilistic record linkage was used, which estimates the probability that a pair of records from different datasets relates to the same person. Additional variables, in particular information on the spouse and family structure were used in the process. Based on probability weights, possible matches were accepted or rejected. Linkage was automated using the Generalized Record Linkage System (GRLS) packaged developed by Statistics Canada. Of all individuals registered in the 1990 census, 81.9% could be linked to a 2000 census record, and during the period 1990-2000, 2.6% to an emigration record and 8.6% to a mortality record. For the remaining individuals with a 1990 census record (6.9%) no satisfactory link could be found. Note also that of all the death recorded from 1990 to the end of 2000, 95.3% could be linked successfully to a census record. |
| **United Kingdom**  Johnson, B., & Langford, A. (2010). Intercensal denominators-feasibility of using the Labour Force Survey to estimate mortality rates by NS-SEC. Health Statistics Quarterly, 45 3-27. | Reduced  NS-SEC (occupation and employment) | 2001-2007 | Mortality follow-up of the national Labor Force Survey | Unlinked approach  In the UK, mortality rates by socio-economic position are preferably estimated by combining death records (numerator) with mid-year population estimates (denominator) obtained at every census (i.e. every 10 years). The method involves the correction for biases resulting from the use of unlinked data. In order to contribute to the monitoring of health inequalities over time, the Office for National Statistics investigated the feasibility of using survey data – namely, the Labour Force Survey (LFS) – to provide population denominators for the estimation of mortality rates by socio-economic status. The LFS collects detailed data on occupation for a sample of approximately 60,000 households in the UK quarterly and annually (annual dataset being boosted). The survey measures socio-economic position by the means of the National Statistics Socio-economic Classification (NS-SEC), which is derived from an individual’s occupation and employment status and the size of their organization. Since size of organisation is not collected on the death register, a version of NS-SEC is used which is derived from occupation and employment status alone. This is known as ‘reduced NS-SEC’. A specific methodology was developed for estimating mi-year populations by age (5-years classes) and NS-SEC using the LFS. |
